# Incidental cardiac fibroma as first manifestation of naevoid basal cell carcinoma syndrome

**DOI:** 10.1093/ehjcr/ytad634

**Published:** 2023-12-19

**Authors:** Roberto Tarantini, Stefano Alonge, Lorenzo Acone, Marco Foti, Giulia Magrini, Andrea Mortara

**Affiliations:** Department of Cardiology, IRCCS Polyclinic San Matteo Hospital, Viale Camillo Golgi, 19, 27100 Pavia, Italy; Department of Molecular Medicine, University of Pavia, Via Forlanini, 14, 27100 Pavia, Italy; Department of Cardiology, IRCCS Polyclinic San Matteo Hospital, Viale Camillo Golgi, 19, 27100 Pavia, Italy; Department of Molecular Medicine, University of Pavia, Via Forlanini, 14, 27100 Pavia, Italy; Department of Cardiology, IRCCS Polyclinic San Matteo Hospital, Viale Camillo Golgi, 19, 27100 Pavia, Italy; Department of Molecular Medicine, University of Pavia, Via Forlanini, 14, 27100 Pavia, Italy; Department of Cardiology, IRCCS Polyclinic San Matteo Hospital, Viale Camillo Golgi, 19, 27100 Pavia, Italy; Department of Molecular Medicine, University of Pavia, Via Forlanini, 14, 27100 Pavia, Italy; Department of Cardiology, IRCCS Polyclinic San Matteo Hospital, Viale Camillo Golgi, 19, 27100 Pavia, Italy; Department of Molecular Medicine, University of Pavia, Via Forlanini, 14, 27100 Pavia, Italy; Department of Cardiology, Polyclinic of Monza, Monza, Italy

**Figure:**
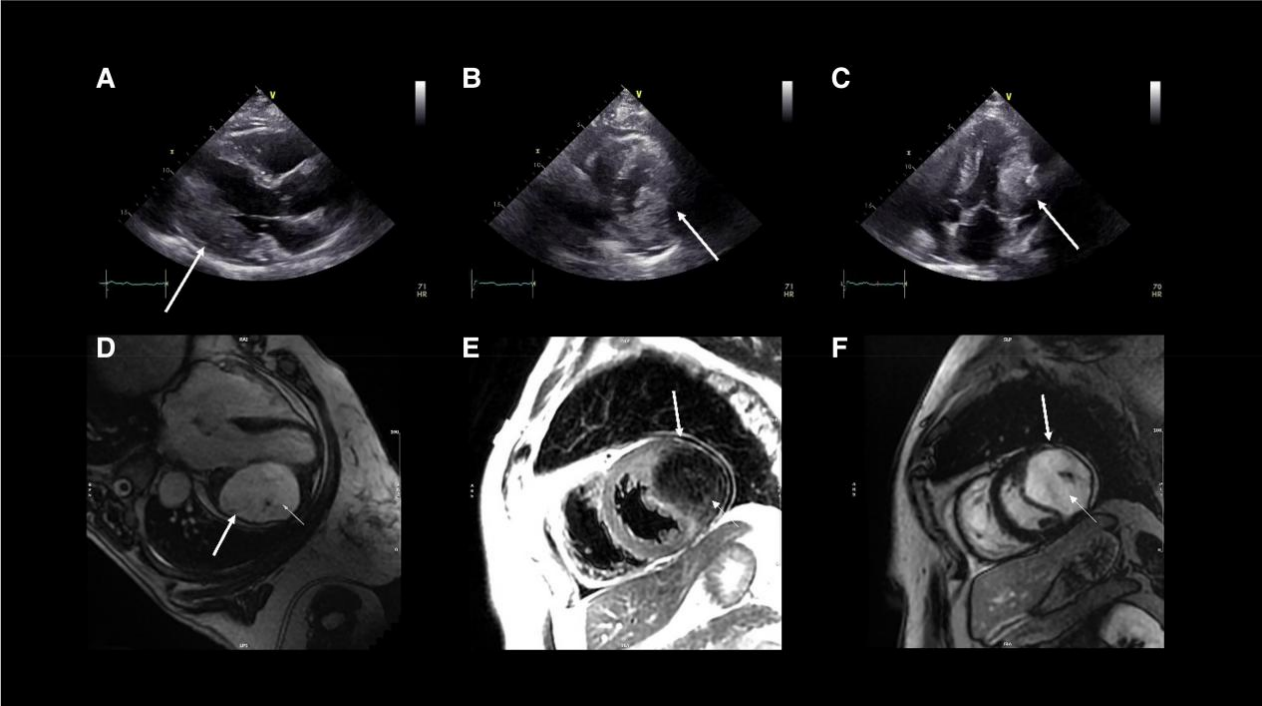


The patient was a 59-year-old woman without comorbidities or history of cardiovascular diseases who underwent a right knee arthroplasty in our hospital. Her electrocardiogram showed sinus rhythm with inverted T-waves in lateral leads. The patient underwent transthoracic echocardiography that revealed a large mass arising from the basal and the middle segments (*Panels A, B, and C*, Supplementary material online, *Movies S1* and *S2*) of left ventricle lateral wall with a preserved left ventricular ejection fraction and no other valvular abnormality. To characterize the mass, cardiac magnetic resonance (CMR) was performed, which demonstrated an intramyocardial mass with well-defined borders and several calcifications. The mass appeared hypointense on T1-weighted and T2-weighted images and showed delayed contrast hyper-enhancement with hypoenhancing central cores (*Panels D, E, and F*, Supplementary material online, *Movies S3* and *S4*). Based on these imaging features, the mass was suggestive of cardiac fibroma with calcifications. Molecular genetic testing identified a heterozygous variant of the PTCH1 gene, pathognomonic for naevoid basal cell carcinoma syndrome (NBCCS). Consequently, the case was discussed with cardiac surgeons: the patient was asymptomatic with no heart failure signs or conduction abnormalities; the surgical risk was very high for position, size, and wall infiltration of the mass; therefore, surgery was excluded and the patient was discharged with follow-up.

Cardiac fibromas are benign tumours typically associated with NBCCS; however, their presence can increase the risk of heart failure, ventricular arrhythmias, and sudden cardiac death. Echocardiography and CMR can better enable clinicians to detect cardiac fibromas in time and to determine the best treatment plan.

## Supplementary Material

ytad634_Supplementary_Data

## Data Availability

No new data were generated or analysed in support of this research.

## Supplementary material

**Consent:** The authors confirm that written consent for the submission and publication of this case, including images, has been obtained from the patient and her parents in line with the Committee on Publication Ethics (COPE) guidance.

**Funding**: None declared.

